# Robotic Sigmoidectomy With Natural Orifice Specimen Extraction: A Single-Center Experience

**DOI:** 10.7759/cureus.49902

**Published:** 2023-12-04

**Authors:** Naved Salim, Camryn Daidone, Leslie Smith, Ahsan Raza

**Affiliations:** 1 Department of Research, Edward Via College of Osteopathic Medicine, Monroe, USA; 2 Department of General Surgery, Rapides Regional Medical Center, Alexandria, USA

**Keywords:** laparoscopic, sigmoid colon, robotic, sigmoidectomy, natural orifice specimen extraction

## Abstract

Background

Natural orifice specimen extraction (NOSE) involves the removal of specimens through a naturally occurring orifice, such as the anus, rather than trans-abdominal extraction. NOSE procedures have been shown to significantly reduce postoperative complications and improve healing.

Objective

The purpose of this case series is to report the outcomes of 27 patients undergoing sigmoidectomies through natural orifice specimen extraction.

Materials and methods

We carefully recorded demographic data on age and BMI, as well as operative data on surgical indication, and length of stay. We also collected data on postoperative complications, including infection, hernia, wound dehiscence, urinary tract infections (UTIs), or anastomotic leaks.

Results

Our patients were majority female (*n* = 21, 77.8%) with a median age of 53.5 (range: 25-79) and median BMI of 33.2 kg/m^2^ (range: 16.7 - 48.3 kg/m^2^). Thirteen patients (48.1%) were obese (BMI > 30.0 kg/m^2^). The majority of these patients underwent sigmoidectomies for benign conditions such as recurrent diverticulitis (*n* = 9, 33.3%), rectal prolapse (*n* = 8, 29.6%), perforated diverticulitis (*n* = 3, 11.1%), colovesical fistula (*n* = 3, 11.1%), and abdominal abscess (*n* = 3, 11.1%) (Table 1). One patient was receiving treatment for sigmoid cancer. The average estimated blood loss was 63.26 mL. The average hospital stay was 3.61 days. Three patients (11.1%) developed a fever postoperatively (temperature >= 100.4 F), which resolved the day after. One patient completed a post-operative hospital stay of 19 days for dialysis and rehab placement. No patients (0.0%) experienced any postoperative complications, including wound infection, hernia, dehiscence, UTIs, or anastomotic leakages. There was no postoperative mortality.

Conclusions

Our study demonstrates the practicality and safety of NOSE procedures for sigmoidectomies as an alternative to transabdominal approaches to treat benign colon diseases.

## Introduction

Natural orifice specimen extraction (NOSE) procedures have been gaining popularity as a minimally or “non-invasive” alternative to transabdominal or even traditional laparoscopic surgery. Traditionally, colon resections have been performed through an open abdomen with a full laparotomy. Laparoscopic techniques were then developed to reduce postoperative infection and improve healing. Studies have shown that laparoscopic sigmoidectomies are superior to open sigmoidectomies in regard to decreasing postoperative mortality and morbidity [1]. Moving further, robotic colon resections were introduced that offered even more favorable post-surgical outcomes. However, both laparoscopic and robotic colon resections require an additional Pfannenstiel incision for the extraction of specimens. Wound infections and intraperitoneal infections can be attributed to this “mini-laparotomy” especially when considering that NOSE procedures have been shown to significantly reduce these complications [2].

During a NOSE procedure, surgical specimens are removed through a naturally occurring opening to the outside of the body such as the anus or the vagina, negating the need for an additional “mini-laparotomy” via a Pfannensteil incision. The NOSE approach is associated with favorable intraoperative and postoperative outcomes, such as less blood loss, shorter surgical stays, lower postoperative pain, and fewer instances of infection and other complications [3]. Initially, there was concern about the additional introduction of bacteria into the peritoneum, increased inflammatory responses, and postoperative outcomes associated with NOSE procedures [4]. Now, there is increasing evidence to support that NOSE approaches are effective in reducing the postoperative complications associated with conventional robotic surgeries involving a fifth incision [5,6]. For example, NOSE procedures are associated with shorter recovery times, less postoperative analgesic use, improved cosmesis and body image, and less intraoperative bleeding [5]. Patients undergoing NOSE procedures have reported a better quality of life than patients undergoing conventional laparoscopic procedures, and the decreased postoperative complications may ultimately lead to decreased hospital stays and healthcare costs.

Natural orifice specimen extraction procedures are common in colorectal surgery for the extraction of benign and malignant sigmoid and rectal masses; however, they are also used for the extraction of various specimens through the vagina [6]. In addition to the transvaginal hysterectomy, which is the gold standard for hysterectomies [7], NOSE procedures have been performed for the removal of large organs such as the kidney, liver, stomach, adrenal glands, and bladder. Procedures involving the removal of such specimens through the vagina as opposed to an additional abdominal incision are associated with reduced postoperative pain and improved cosmesis with no increase in postoperative complications [6].

Current literature studying the efficacy and potential benefits of NOSE procedures over transabdominal or conventional laparoscopic surgeries in colorectal surgery suggests that even though NOSE procedures may have longer operative times, they generally produce favorable outcomes such as lower postoperative pain, shorter length of hospital stay, and earlier return of bowel function [3,8]. However, some studies suggest that these favorable outcomes may be associated with specific characteristics such as patient body mass index (BMI) <30, American Society of Anesthesiologists (ASA) class <3, and specimen diameter <6.5 cm [8]. Other studies suggest that NOSE procedures may provide significant benefits for obese patients due to their less invasive nature [9-11].

In this case series, we aim to report on the surgical outcomes of performing robotic sigmoidectomies using NOSE methods through the anus for the treatment of predominantly benign colon conditions. We hope that this study will contribute to a body of research seeking to evaluate surgical outcomes of sigmoidectomies using NOSE procedures.

## Materials and methods

From May 2020 to August 2023, there were 27 cases of NOSE sigmoidectomy procedures performed by a single surgeon at Rapides Regional Medical Center in Alexandria, LA. We carefully recorded demographic data on age and BMI, as well as operative data on surgical indication and length of stay. We also collected data on postoperative complications, including infection, hernia, wound dehiscence, or anastomotic leaks.

Surgical procedure

**Figure 1:**
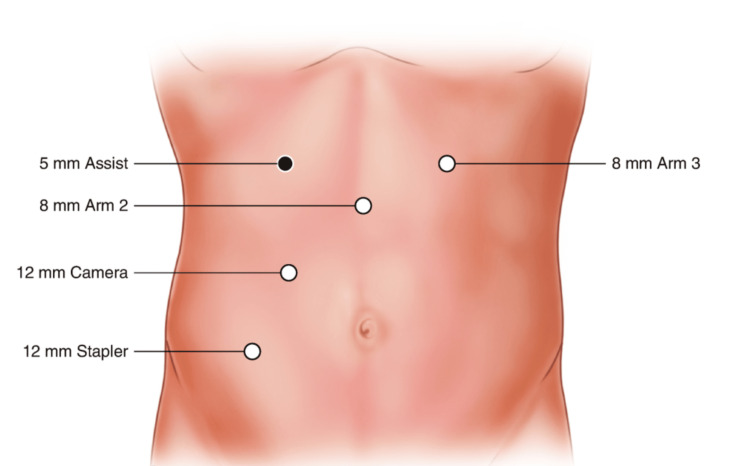
Robotic sigmoidectomy port placements Reference: Themes 2020 [12]

**Figure 2:**
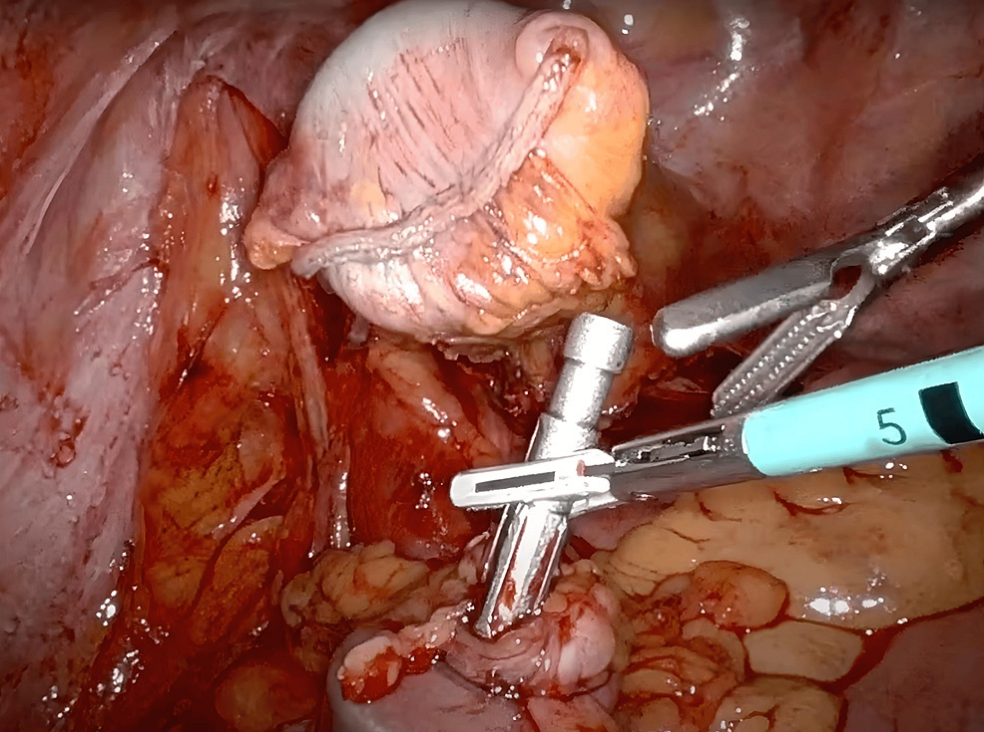
A crucial step involving the anastomosis of the two parts of the colon. The anvil arm is shown on the bottom, protruding from the proximal colon section. The stapled "rectal stump" is shown at the top of the figure.

**Figure 3:**
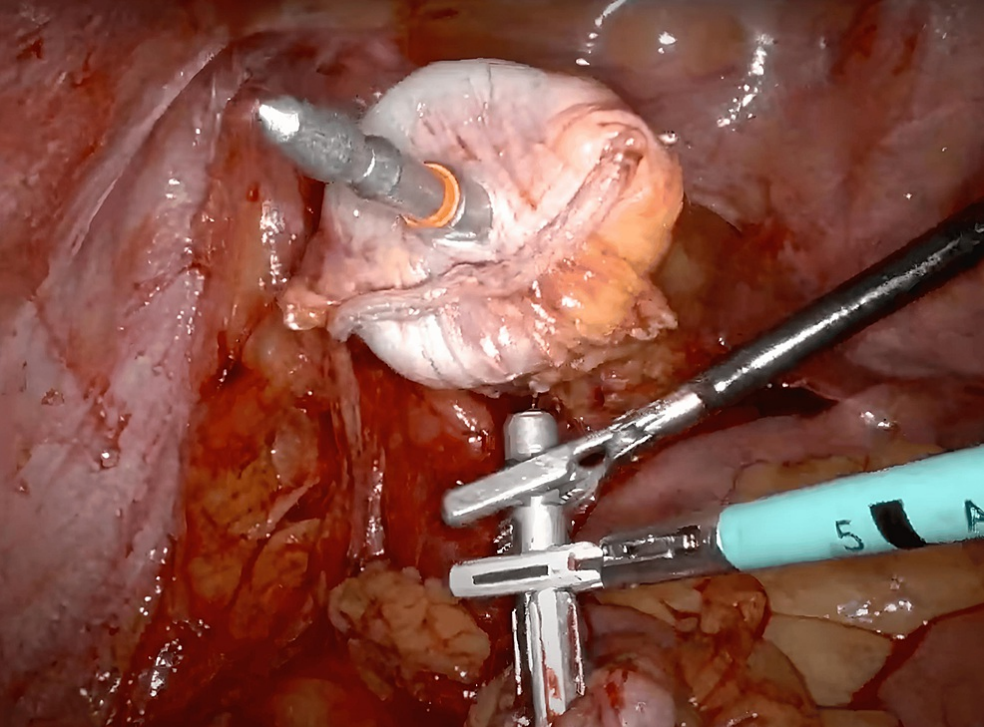
A pin is inserted through the rectal stump (top of the figure) and the anvil (bottom of the figure) is placed on the pin.

**Figure 4:**
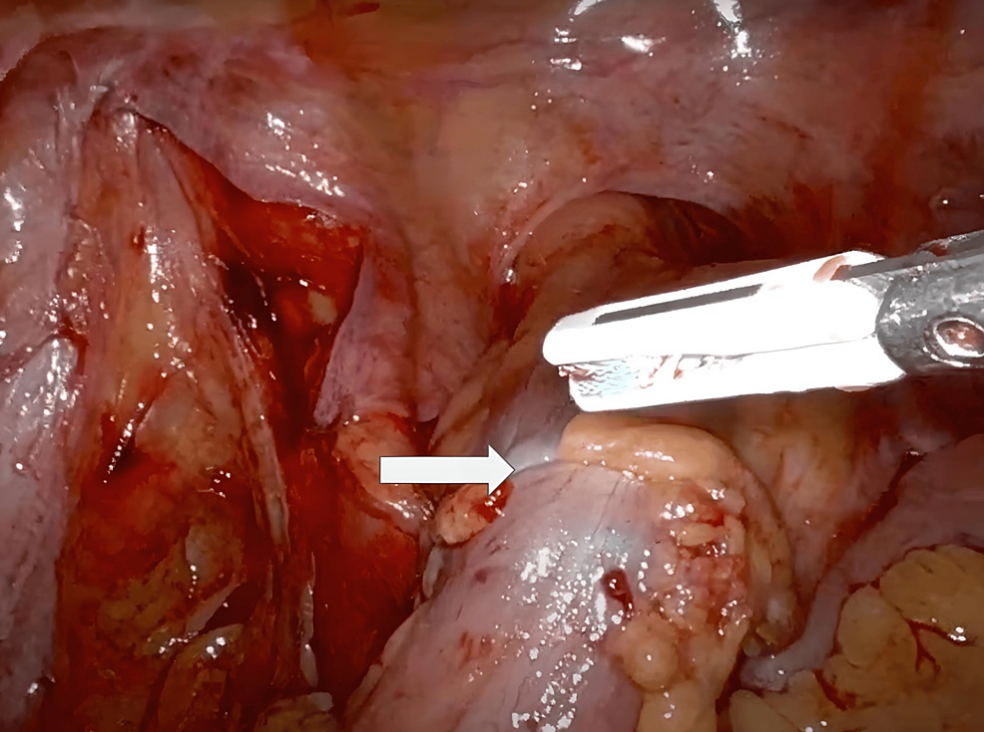
Final anastomosis of the two colon sections (arrow)

The patient was placed in a modified lithotomy position after general endotracheal anesthesia was induced and a Foley catheter was placed. After sterile prepping, all port sites and the extraction site were injected with a Marcaine solution into the skin, subcutaneous, and peritoneal levels. A 15-blade was used to make an 8 mm left upper quadrant incision. A 5 mm Visiport (Medtronic plc, Dublin, Ireland) was atraumatically inserted into the abdomen. The abdomen was insufflated to 15 mmHg pressure. Under direct vision, two 8 mm or 12 mm ports were placed in the left upper quadrant and the right lower quadrant, a 12 mm port in the right lower quadrant, and a 5 mm AirSeal assist port (CONMED, Utica, New York, USA) in the right mid-abdomen. The original 5 mm Visiport was switched out for an 8 mm robotic port (Figure 1). While still in the lithotomy position, the patient was tilted toward the right so that the small bowel could be swept out of the way. With the robot docked and instruments inserted atraumatically, the peritoneum overlying the inferior mesenteric artery was divided. The left ureter was identified and swept posteriorly. This vessel was skeletonized and then a 30 mm robotic vascular staple load was used to divide the inferior mesenteric artery. The left colic artery was taken with the vessel sealer and retroperitoneal dissection was carried out laterally. The descending colon was mobilized along the white line of Toldt. Sharp dissection with cautery scissors and blunt dissection was used to carefully separate the sigmoid colon off of the anterior pelvic peritoneum and this dissection was carried down distally to the rectosigmoid junction. The vessels were skeletonized and the mesorectum was divided with the vessel sealer. The descending colon was then mobilized up to the splenic flexure and the splenic flexure was mobilized. The splenocolic ligament was fully divided for full mobilization of the splenic flexure medially. The mesentery was then taken up to a soft and pliable portion of the descending colon, and the marginal artery was taken with the vessel sealer. Indocyanine green (ICG) was injected and Firefly® was activated, which showed a clear demarcation point at the descending colon junction and rectosigmoid junction. The rectosigmoid junction was brought up and divided with one firing of a 60 mm green robotic staple load. The staple line of the rectal stump was then excised with monopolar scissors. The assistant inserted the ring forceps into the rectum and placed the anvil inside the abdominal cavity. A small enterotomy was made distal to the proximal staple line site, and the anvil was inserted into the colon (Figure 2). The enterotomy was closed with a V-lock suture. A 60 mm green load stapler was used and fired across the colon section proximal to the rectosigmoid junction. With the help of monopolar scissors, the anvil was gently introduced right by the staple line. The specimen was retrieved through the anal orifice (Figure 3). The assistant surgeon performed a saline leak test on the rectal stump which came out negative. The assistant surgeon then placed a stapler into the rectal stump. The pin was brought out just anterior to the staple line. The anvil was placed on the pin (Figure 3), and the anastomosis was tightened down and fired after assuring anatomic orientation with no twisting of the left colon mesentery (Figure 4). The stapler was removed, and two intact tissue donuts were found in the stapler. Proctoscopy and a saline leak test were performed, which were both negative. ICG was re-dosed and Firefly® was activated again which showed excellent blood flow to the proximal and distal ends of the colorectal anastomosis. A thorough abdominal and pelvic irrigation with warm saline was performed, and the abdomen was found to be hemostatic. A Carter-Thomason needle was used to place 0 Vicryl suture through the 12 mm port site fascia, and this was tied down. The robotic and laparoscopic instruments were removed. The subcutaneous tissue was irrigated well with saline, and then 4-0 Monocryl subcuticular sutures were used to close the skin. A Dermabond dressing was applied to the wound, and the patient was extubated and taken to the recovery room in stable condition. All needle, instrument, and sponge counts were correct.

## Results

From May 2020 to August 2023, 27 patients underwent robotic transrectal NOSE in the Department of Surgery at Rapides Regional Medical Center in Alexandria, Louisiana. The majority of our patients were female (n = 21, 77.8%) with a median age of 53.5 (range: 25-79) and median BMI of 31.2 kg/m^2^ (range: 16.7 - 48.3 kg/m^2^). Fifteen patients (55.56%) were obese (BMI > 30.0 kg/m^2^). The majority of these patients underwent sigmoidectomies for benign conditions such as recurrent diverticulitis (n = 9, 33.3%), rectal prolapse (n = 8, 29.6%), perforated diverticulitis (n = 3, 11.1%), colovesical fistula (n = 3, 11.1%), perforated diverticulitis with abdominal wall abscess (n = 3, 11.1%) (Table 1). The patients with a colovesical fistula had their fistulas sutured closed on the bladder and a Foley catheter placed, in addition to the sigmoidectomy. One patient was receiving treatment for sigmoid cancer. This patient received chemoradiotherapy six weeks before surgery, waiting one month between the last treatment and the actual operation. The average estimated blood loss was 63.27 mL. The average hospital stay was 2.61 days and the median hospital stay was one day. Three patients (11.1%) developed a fever postoperatively (temperature >= 100.4 F), which resolved the day after. One patient completed a postoperative hospital stay of 19 days for dialysis and waiting for rehab placement. No patients (0.0%) experienced postoperative complications under the Clavien-Dindo grades, including wound infection, hernia, dehiscence, or anastomotic leakages. There was no postoperative mortality.

**Table 1:** Surgical indications of 27 patients undergoing robotic transanal NOSE NOSE: natural orifice specimen extraction

Indication for Surgery	Number of Patients
Recurrent Diverticulitis	10 (37.1%)
Rectal Prolapse	8 (29.6%)
Perforated Diverticulitis	3 (11.1%)
Perforated Diverticulitis w/ Abdominal Wall Abscess	3 (11.1%)
Colovesical Fistula	3 (11.1%)

The average BMI for our patient population was 31.69 kg/m^2^ (range: 16.7 - 48.3). Fifteen patients had a BMI greater than 30 kg/m^2^ (obese) and the rest of the 12 patients had a BMI less than 30 kg/m^2^ (non-obese). Of the 15 obese patients, two of them (13.33%) developed postoperative fever. Of the 12 non-obese patients, one of them (8.33%) developed a postoperative fever. The difference between these two values is 5%. By calculating a Z-score for two population proportions, we obtained a Z-score of 0.4108 and a p-value of 0.6818. With a confidence interval of 95% (alpha = 0.05), our calculated p-value is greater than 0.05. Therefore, there is no significant difference between the occurrence of postoperative fever in obese patients and non-obese patients. There were no other complications noted in any of the patients.

## Discussion

A recent meta-analysis of 21 randomized control trials analyzing 2,112 patients evaluating NOSE procedures for transanal colorectal cancer extraction indicated that NOSE procedures were associated with longer operative time, less blood loss, shorter hospital stays, shorter time until passage of flatus, improved cosmetic scores, better pain score, and fewer postoperative complications and infections [3]. Another meta-analysis of 1787 patients undergoing transanal extraction of colorectal tumors showed favorable outcomes for NOSE procedures such as decreased post-surgical complications, improved recovery of GI function, and decreased use of postoperative analgesics [4].

In this study, we intended to evaluate the outcomes of robotic sigmoidectomy via NOSE methods in patients with predominantly benign sigmoid colon diagnoses. Of our 27 patients who underwent sigmoidectomy with NOSE, none of these patients experienced postoperative infections, hernia, wound dehiscence, or anastomotic leakages. None of these patients underwent conversion to an open procedure. The average hospital stay for these patients was 2.61 days. One patient completed a 19-day hospital stay due to the patient needing dialysis and waiting for a rehab placement. Excluding this patient, whose extended stay was due to factors unrelated to this procedure, the average length of stay was two days.

Three patients developed a fever postoperatively (temperature >= 100.4 F), which resolved the day after. No significant similarities were made between these patients. Two of these patients were females aged 63 and 71 and the third patient was a 37-year-old male. The 63-year-old female had a BMI of 16.6 kg/m^2^ and an estimated blood loss of 50 mL. The 71-year-old female had a BMI of 31.2 kg/m^2^ and an estimated blood loss of 150 mL. The 37-year-old male patient had a BMI of 48.3 kg/m^2^ with an estimated blood loss of 200 mL. These characteristics may have contributed to the development of fever due to increased blood loss compared to the average estimated blood loss (63.27 mL). These patients also had either a higher or lower BMI compared to the average BMI of 31.69 kg/m^2^ and median BMI of 31.2 kg/m^2^. Their BMI values were classified as either overweight or underweight, which may have also made them susceptible to developing a fever due to unhealthy body habitus. Surgical site infection is the most common postoperative complication after surgery. This complication has been associated with negative economic impact, increased morbidity, extended postoperative hospital stay, readmission, sepsis, and death [13,14]. None of these patients met the criteria for surgical site infection, postoperative infection, or sepsis.

We decided to compare whether the complication of postoperative fever was more prominent in obese patients versus non-obese patients. Of the 15 obese patients, two of them developed postoperative fever. Of the 12 non-obese patients, one of them developed a postoperative fever. A Z-score for two population proportions was calculated at 0.4108, and our calculated p-value was 0.6818. With a confidence interval of 95% (alpha = 0.05), we determined that there was no significant difference between the occurrence of postoperative fever in obese patients and non-obese patients. There were no other complications noted in any of these patients. While some studies suggest that the ideal candidate to receive a NOSE procedure should have a BMI of <30 [4], our study suggests that the option for robotic NOSE sigmoidectomy procedures should not be limited to patients with a BMI <30 as provider training and comfort permits. Our sample size of patients was relatively small (n = 27) so future studies may explore if these conclusions remain consistent within larger sample sizes.

NOSE procedures likely allow for a safer alternative to trans-abdominal specimen extraction procedures. A systematic review of 14 randomized controlled trials using NOSE for the treatment of colorectal cancer showed that the NOSE procedures were significantly superior to conventional laparoscopic approaches in several safety outcomes, including fewer surgical site infections and fewer perioperative complications such as ischemia, bleeding, anal dysfunction, and ileus. Other safety outcomes, such as the presence of intra-abdominal abscess, anastomotic leakage, and blood loss, showed no significant difference between NOSE procedures and the conventional laparoscopic approach [15]. Our study supports these findings and suggests that NOSE procedures are safe and may provide additional safety benefits compared to conventional laparoscopic approaches.

Our analysis shows the outcomes of patients undergoing NOSE procedures for primarily benign disease of the colon with the most common indication for surgery being diverticulitis. Future studies can continue to evaluate the outcomes of NOSE procedures in a variety of procedures. NOSE colectomies have been successful in treating other conditions, including endometriosis of the bowel [16,17] and colorectal cancers [15]. The treatment of malignant conditions using NOSE procedures is increasingly common though there is the concern for tumor seeding into the peritoneum. Many studies have shown that NOSE procedures are associated with favorable outcomes in the treatment of malignant colorectal disease, however, exposing the rectum to the peritoneal cavity in the case of colorectal cancer poses additional risks that warrant further investigation [18].

The involvement of only one surgeon is a significant strength of our study. This ensured a standardized NOSE procedure across all patients with no potential variations that may arise from procedures performed by different surgeons at different institutions. However, this also brought forth a limitation of our study involving a small sample size from a single surgeon at a single institution. Future studies may include larger sample sizes involving patients from different surgeons across multiple institutions.

## Conclusions

Our retrospective analysis of 27 patients at a single institution highlighted the postoperative outcomes of performing robotic natural orifice specimen extraction procedures. Overall, it was noted that this procedure was safe and effective for our patients, resulting in favorable outcomes in the context of postoperative stay, urinary tract infection, anastomotic leakages, wound infection, herniation, and dehiscence.

Although conventional trans-abdominal colon extractions remain the more widely used procedure, our study demonstrates the practicality and safety of NOSE procedures for sigmoidectomies for benign colon diseases.
